# Changing effect of the numerator–denominator bias in unlinked data on mortality differentials by education: evidence from Estonia, 2000–2015

**DOI:** 10.1136/jech-2020-214487

**Published:** 2020-07-20

**Authors:** Domantas Jasilionis, Mall Leinsalu

**Affiliations:** 1 Laboratory of Demographic Data, Max Planck Institute for Demographic Research, Rostock, Germany; 2 Demographic Research Centre, Vytautas Magnus University, Kaunas, Lithuania; 3 Stockholm Centre for Health and Social Change, Södertörn University, Huddinge, Sweden; 4 Department of Epidemiology and Biostatistics, National Institute for Health Development, Tallinn, Estonia

**Keywords:** Mortality, Inequalities, Education, Measurement, Eastern Europe

## Abstract

**Background:**

This study highlights changing disagreement between census and death record information in the reporting of the education of the deceased and shows how these reporting differences influence a range of mortality inequality estimates.

**Methods:**

This study uses a census-linked mortality data set for Estonia for the periods 2000–2003 and 2012–2015. The information on the education of the deceased was drawn from both the censuses and death records. Range-type, Gini-type and regression-based measures were applied to measure absolute and relative mortality inequality according to the two types of data on the education of the deceased.

**Results:**

The study found a small effect of the numerator–denominator bias on unlinked mortality estimates for the period 2000–2003. The effect of this bias became sizeable in the period 2012–2015: in high education group, mortality was overestimated by 23–28%, whereas the middle education group showed notable underestimation of mortality. The same effect was small for the lowest education group. These biases led to substantial distortions in range-type inequality measures, whereas unlinked and linked Gini-type measures showed somewhat closer agreement.

**Conclusions:**

The changing distortions in the unlinked estimates reported in this study warn that this type of evidence cannot be readily used for monitoring changes in mortality inequalities.

## INTRODUCTION

Monitoring socioeconomic inequalities in mortality is a crucial component for designing appropriate policies promoting more sustainable health development.^1 2^ However, producing reliable evidence about the magnitude and changes in mortality inequalities requires precise register-based or census-linked data. Such data covering entire populations are still missing for many developed countries. A widely used alternative in these cases is relying on cross-sectional unlinked data based on separate tabulations of deaths and population exposures by socio-economic groups. The major problem with unlinked data is the disagreement between the sources of information on death and census records.^3–^ ^6^ The socio-demographic information provided on death certificates is considered as lower quality due to a higher probability of misreporting by proxy informants.^7 8^ The mismatch in the sources of information establishing numerators and denominators of death rates may lead to distortions of aggregated mortality and inequality estimates. Matching studies checking the validity of socio-demographic information on death records are scarce.^9–^ ^13^ To our knowledge, the only evidence on the importance of numerator–denominator bias in Eastern Europe comes from two studies on Lithuania.^10 13^ These studies found a substantial misreporting of education and ethnicity on death records leading to biases in group-specific mortality and failing to report the gradient of inequality correctly.

This study extends prior evidence about the numerator–denominator bias in unlinked data by providing new evidence based on the data for Estonia with a special focus on the change in the size of the bias in time. In addition, the current study broadens the scope of previous analyses by performing systematic sensitivity testing of a wider range of inequality measures.

## DATA AND METHODS

This study uses an aggregated census-linked mortality dataset provided by Statistics Estonia. These data were compiled from longitudinal mortality follow-up studies based on the 2000 and 2011 censuses. All permanent residents of Estonia taking part in both censuses were followed from the census dates (31 March 2000 and 31 December 2011) until the date of death or end of the follow-up period (31 December 2003 and 31 December 2015, respectively). Of all death records, 95–98% were successfully linked to the preceding censuses. For the analyses, the data were organised into two periods (2000–2003 and 2012–2015). The age-specific population exposures by education used to calculate both census-linked and unlinked mortality estimates were estimated by aggregating person-years lived by individuals during the period of observation (also accounting for the change in exact age within each year of observation). Meanwhile, deaths were grouped according to the exact age at death.

For linked estimates, education of the deceased was derived from the census at the beginning of follow-up. For unlinked estimates, education of the deceased stemmed from death records. For both linked and unlinked estimates, education-specific person-years of exposure were calculated according to the census information on education and subsequent follow-up information. The original educational coding in these variables was reclassified using the three broad International Standard Classification of Education (ISCED)11 categories: (1) *low* education combining primary and lower secondary education (ISCED11 categories 0–2); (2) *middle* education combining upper secondary and post-secondary non-tertiary education (ISCED11 categories 3–5); (3) *high* education referring to tertiary education (ISCED11 categories 6–8). For a better match with death records, ISCED11 category 5 was combined with middle education. The per cent of missing education was very low for both the census and death record information (0–0.8%) with the exception of unlinked deaths for the period 2012–2015 (missing education was observed for 13% of death records for males and 14% of death records for females). For this period, deaths and person-years of exposure with unknown education were redistributed using a conservative approach assuming a proportional distribution across the three educational categories (online annex table 1). In all the remaining cases, negligible numbers of deaths and exposures with unknown education were excluded from the analyses.

10.1136/jech-2020-214487.supp1 Supplementary data

Education-specific mortality for males and females was measured by age-standardised death rates (SDRs) using the WHO European Population (1976) as a standard. Relative mortality differences were assessed using age-adjusted Poisson regression mortality rate ratios (MRRs). More advanced numerically calculated inequality measures (average intergroup difference (AID) and Gini coefficient)) were applied to account for the total amount of inequality across all educational groups and group-specific weights in the population (online annex table 2).^14 15^ Regression-based inequality measures (Slope Index of Inequality (SII) and Relative Index of Inequality (RII)) were calculated using common algorithm described by Anand *et al.* ^14^ The public health impact of inequality was estimated using population-attributable fractions (PAFs).^14^

## RESULTS

Table 1 provides aggregated education-specific mortality estimates by education given on census and death records in Estonia in the periods 2000–2003 and 2012–2015. The results reveal quite a small effect of the numerator–denominator bias on unlinked mortality estimates in the first period and a pronounced discrepancy between the linked and unlinked education-specific mortality estimates in the second period. The high education group showing overestimation of mortality by 23–28% in the period 2012–2015 was the most affected. Meanwhile, the unlinked SDRs for males and females with middle education for the same period were affected by the underestimation of mortality. The most striking case concerns females at age 65 with middle education in the period 2012–2015 showing lower mortality than among females with high education. The discrepancies were surprisingly small for the lowest education group except for females aged 30–64 years in the period 2012–2015 (table 1).

**Table 1:** Linked and unlinked mortality estimates by education group for males and females in Estonia, 2000–2003 and 2012–2015

	Males				Females			
	Linked		Unlinked		Linked		Unlinked	
	2000–2003	2012–2015	2000–2003	2012–2015	2000–2003	2012–2015	2000–2003	2012–2015
SDR (30+ years)								
High	1512	1078	1498	1282	885	612	864	795
Middle	2243	1667	2385	1588	1095	779	1159	703
Low	3113	2435	3048	2496	1560	1217	1519	1288
SDR (30–64 years)								
High	549	340	529	448	234	155	232	208
Middle	1122	735	1167	683	382	261	390	228
Low	1789	1295	1768	1404	722	554	698	644
SDR (65+ years)								
High	5538	4164	5548	4769	3607	2526	3510	3251
Middle	6932	5565	7475	5373	4076	2942	4375	2689
Low	8648	7201	8399	7062	5063	3990	4951	3978

SDR, age-standardised death rate per 100 000 person-years.

The observed biases in education-specific unlinked mortality estimates predetermined substantial distortions in the corresponding range-type measures of mortality inequality (table 2). For both males and females aged 30 years and over, the maximal absolute difference in SDRs according to the unlinked data was significantly underestimated, especially for females in the second period. Meanwhile, MRRs were quite similar for the period 2000–2003 and remarkably different for the period 2012–2015. The most significant distortion in the unlinked MRRs was observed for females with middle education leading to the artificial advantage of this group against the highest education group. In all the remaining cases, the MRRs based on unlinked data for 2012–2015 were notably lower than those derived using linked data.

**Table 2:** Linked and unlinked estimates of mortality inequality by education for males and females in Estonia, 2000–2003 and 2012–2015

	Males				Females			
	Linked		Unlinked		Linked		Unlinked	
	2000–2003	2012–2015	2000–2003	2012–2015	2000–2003	2012–2015	2000–2003	2012–2015
Age: 30+ years								
*Maximal difference in SDRs*	1601	1357	1550	1214	675	605	655	493
*Mortality rate ratio*								
High (ref.)	1.00	1.00	1.00	1.00	1.00	1.00	1.00	1.00
Middle	1.48	1.55	1.59	1.24	1.24	1.27	1.34	0.88
Low	2.06	2.26	2.04	1.95	1.76	1.99	1.76	1.62
*AID*	283.7	235.1	262.8	209.1	126.6	101.8	117.6	98.8
*Gini (×100)*	12	14	11	12	11	13	10	12
*SII*	2111	1514	1897	1284	820	574	699	404
*RII*	2.50	2.54	2.25	2.17	2.00	2.04	1.80	1.63
*PAF (%)*	36	37	38	25	26	25	29	5
Age: 30–64 years								
*Maximal difference in SDRs*	1240	955	1239	956	489	399	467	436
*Mortality rate ratio*								
High (ref.)	1.00	1.00	1.00	1.00	1.00	1.00	1.00	1.00
Middle	2.04	2.16	2.21	1.52	1.63	1.69	1.68	1.10
Low	3.26	3.81	3.34	3.13	3.09	3.58	3.02	3.10
*AID*	201.0	151.4	199.0	145.2	69.5	51.3	67.3	41.4
*Gini (×100)*	17	20	17	19	17	20	17	16
*SII*	1364	982	1311	919	455	322	435	253
*RII*	3.62	4.94	3.40	4.28	3.56	4.49	3.33	3.01
*PAF (%)*	53	54	56	40	41	38	42	19
Age: 65+ years								
*Maximal difference in SDRs*	3109	3037	2851	2294	1457	1463	1441	728
*Mortality rate ratio*								
High (ref.)	1.00	1.00	1.00	1.00	1.00	1.00	1.00	1.00
Middle	1.25	1.34	1.35	1.13	1.13	1.16	1.25	0.83
Low	1.56	1.73	1.51	1.48	1.40	1.58	1.41	1.22
*AID*	594.7	631.3	465.8	506.4	278.9	305.4	214.2	311.1
*Gini (%)*	8	11	6	9	6	9	5	10
*SII*	5146	4249	4092	3406	2433	1958	1856	1493
*RII*	2.00	2.12	1.72	1.81	1.71	1.85	1.50	1.59
*PAF (%)*	28	29	28	19	22	22	24	0

AID, average intergroup difference per 100 000 person-years; Gini, intergroup Gini coefficient; PAF, population-attributable fraction; RII, Relative Index of Inequality; SDR, age-standardized death rate per 100 000 person-years; SII, Slope Index of Inequality per 100 000 person-years.

We found that using numerically calculated inequality measures (AID and Gini) accounting for mortality rates and population weights for each educational group may lead to a somewhat closer agreement between the linked and unlinked inequality measures. The biggest difference was detected comparing AID and Gini coefficients for females aged 30–64 years. In this case, underestimation of total mortality variation by education using unlinked data was about 20%. The corresponding disagreement was much lower for males in the same age group and both sexes at ages 30+ and 65+. Interestingly, similar regression-based inequality measures (SII and RII) show more pronounced discrepancies. Our final comparison examining PAFs warns that population-based mortality burden due to educational inequalities estimated according to unlinked data was vastly undercounted in the second period.

## DISCUSSION

The study found that the growing effect of misreporting of education on death records in Estonia had a substantial impact on the decreasing quality of education-specific mortality estimates based on unlinked data. This bias was also responsible for distortions in the magnitude and even direction of change in mortality inequalities. This finding is a warning sign against using unlinked estimates for monitoring changes in mortality inequality. A slightly better agreement was achieved using more advanced numerically calculated Gini-type measures of inequality (except for females aged 30–64 years). The advantage of the AID and Gini coefficient is probably related to a very good agreement between the unlinked and linked SDRs for the lowest educational group showing larger population weights.

The observed distortions in education-specific mortality estimates derived from the unlinked data using death record-based information about education can be attributed to a variety of changeable factors. First, notable discrepancies may occur due to differences in the design and wording of questions on education in both the census and death records.^10^ As in other countries, census questions in Estonia were more detailed and better suited to classify own education within different educational systems functioning during various historical periods. This design contrasts to less detailed questions available on death records. Differently from death records, the census records also specify the entry-level for each educational level. Studies suggest that reported information on death records may depend on the socio-demographic characteristics of proxy informants and the deceased.^5 10^ For example, the Lithuanian study shows that misreporting of education increases with age and is more frequent among those dying from alcohol-related or external causes of death and non-married individuals and Russian, Polish and other ethnic groups.^10^ Although self-reported education in the census is also prone to reporting errors, using the same source of information for both the deceased and population exposures allows to avoid the well-known numerator–denominator bias.^3–^ ^6^

One of the main reasons for the changing bias in the unlinked mortality data for Estonia can be related to the spread of post-secondary non-higher education. It is possible that a substantial share of third-party informants assumed this category being a part of the tertiary (high) education. This misclassification would explain a notable overestimation of mortality in the high education group, as reflected by the unlinked data. Finally, the rise in the proportion of the unknown category from almost 0% to 13–14% in the period 2012–2015 suggests the decreasing quality of filling this information on death records. Applying a simple proportional redistribution of unlinked deaths across the three educational groups is a limitation of the study. However, sensitivity analyses have shown that applying such an assumption leads to more plausible results if compared to the alternative solution based on assigning all deaths with unknown education to the lowest educational category. We were not able to test more sophisticated multiple imputation methods requiring access to the individual-level data. Finally, this study used education to rank socio-economic groups and did not provide any insights into the causal impact of education on mortality.

The results of this study have important implications for interpreting past and emerging evidence on mortality differentials based on unlinked data. Our findings warn that small numerator–denominator bias observed at some point in time cannot guaranty the sustainability of such a pattern in the future. The misreporting of education seems to be country-specific, indicating that the numerator–denominator bias can take different forms in various contexts. This conclusion is supported by completely different evidence from Lithuania for the period 2001–2004, revealing a very important effect of the numerator–denominator bias on education-specific mortality estimates based on unlinked data.^10^ Therefore, the finding suggesting that more advanced Gini-type measures are less prone to the numerator–denominator bias may reflect the Estonian specifics and do not apply to other countries. Scientific and policy efforts should be reinforced by informing policy-makers about the risks of using unlinked data and highlight the need for more reliable evidence based on the registry- or census-linked data.

**What is already known on this subject**

- The numerator–denominator bias leads to notable distortions in education-specific mortality.
- The misreporting patterns and magnitude of the bias in the unlinked data vary across countries.
- Matching studies checking the validity of socio-demographic information on death records are scarce.

**What this study adds**

- The numerator–denominator bias in the unlinked mortality data may change in time, suggesting that such evidence cannot be readily used for monitoring mortality inequalities.
- Conventional range-type measures of inequality are particularly sensitive to the bias.
- High and middle education groups in Estonia were particularly affected by misreporting on death records.
