# Supplementary material for: Changing effect of the numerator–denominator bias in unlinked data on mortality differentials by education: evidence from Estonia, 2000–2015
Source: J Epidemiol Community Health. 2020 Jul 20;75(1):88–91. doi: 10.1136/jech-2020-214487 (PMC7788481; doi:10.1136/jech-2020-214487)
Supplement: Supplementary data [file jech-2020-214487supp001.pdf]

Online Annex Table 1. Deaths and person years of exposure by education. Estonia, 200-2003 and 2012-2015.

	Males						Females					
	2000-2003			2012-2015			2000-2003			2012-2015		
Education	Linked	Unlinked	Person years of exposure	Linked	Unlinked	Person years of exposure	Linked	Unlinked	Person years of exposure	Linked	Unlinked	Person years of exposure
Age 30+												
High	2629 (8.6%	2591 (8.5%)	219653 (16.6%)	3694 (13.5%)	4398 (16.0%)	329650 (22.5%)	1757 (5.6%)	1719 (5.5%)	306052 (17.4%)	3403 (11.2%)	4404 (14.5%)	532652 (28.3%)
Middle	10507 (34.3	11062 (36.1%)	715758 (54.2%)	11618 (42.4%)	11000 (40.1%)	820477 (56.0%)	8696 (27.7%)	9159 (29.2%)	960331 (54.5%)	11214 (36.8%)	10164 (33.4%)	1005511 (53.4%)
Low	17466 *(57.1%)*	16948 (55.4%)	384087 (29.1%)	12108 (44.2%)	12023 (43.8%)	314533 (21.5%)	20907 (66.7%)	20482 (65.3%)	494133 (28.1%)	15853 (52.0%)	15902 (52.2%)	344496 (18.3%)
Total	30602 (100.0	30602 (100.0%)	1319498 (100.0%)	27421 (100.0%)	27421 (100.0%)	1464660 (100.0%)	31360 (100.0%)	31360 (100.0%)	1760517 (100.0%)	30470 (100.0%)	30470 (100.0%)	1882660 (100.0%)
Age 30-64												
High	1020 (8.1	986 (7.8%)	182432 (17.2%)	882 (10.1%)	1158 (13.3%)	259723 (22.6%)	582 (11.1%)	577 (11.0%)	256229 (20.8%)	622 (17.5%)	834 (23.5%)	424773 (33.9%)
Middle	6082 (48.3	6274 (49.9%)	640597 (60.4%)	5254 (60.3%)	4872 (55.9%)	696183 (60.6%)	2919 (55.5%)	2983 (56.7%)	800017 (64.9%)	2229 (62.8%)	1949 (54.9%)	716350 (57.2%)
Low	5484 (43.6	5326 (42.3%)	236814 (22.3%)	2579 (29.6%)	2686 (30.8%)	193071 (16.8%)	1760 (33.5%)	1700 (32.3%)	176454 (14.3%)	696 (19.6%)	764 (21.5%)	111753 (8.9%)
Total	12585 (100.0	12585 (100.0%)	1059844 (100.0%)	8715 (100.0%)	8715 (100.0%)	1148977 (100.0%)	5260 (100.0%)	5260 (100.0%)	1232701 (100.0%)	3547 (100.0%)	3547 (100.0%)	1252877 (100.0%)
Age 65+												
High	1609 (8.9	1606 (8.9%)	37221 (14.3%)	2812 (15.0%)	3240 (17.3%)	69927 (22.2%)	1175 (4.5%)	1143 (4.4%)	49823 (9.4%)	2781 (10.3%)	3570 (13.3%)	107879 (17.1%)
Middle	4425 (24.6	4788 (26.6%)	75160 (28.9%)	6364 (34.0%)	6128 (32.8%)	124294 (39.4%)	5778 (22.1%)	6176 (23.7%)	160314 (30.4%)	8985 (33.4%)	8215 (30.5%)	289161 (45.9%)
Low	11983 (66.5	11623 (64.5%)	147273 (56.7%)	9529 (50.9%)	9338 (49.9%)	121462 (38.5%)	19147 (73.4%)	18782 (72.0%)	317679 (60.2%)	15157 (56.3%)	15138 (56.2%)	232743 (37.0%)
Total	18017 (100.0	18017 (100.0%)	259654 (100.0%)	(18706 (100.0%)	18706 (100.0%)	315683 (100.0%)	26100 (100.0%)	26100 (100.0%)	527815 (100.0%)	26923 (100.0%)	26923 (100.0%)	629783 (100.0%)

Online Annex Table 2. Short descriptions of the Gini-type measures of inter-group mortality inequality by education.

Measure	Description and equation
**Inter-group difference (AID)**	This measure refers to population-weighted average of absolute differences between each pair of education-specific age-standardized death rates (SDRs): $$AID = \frac{1}{2}\sum_{i=1}^{3}\sum_{j=1}^{3}\left\|SDR_i - SDR_j\right\| p_i p_j$$ where p_i and p_j are the weights of each educational category i and j (i, j = 1, 2, 3) in a total population, whereas SDR_i and SDR_j refer to education-specific standardized death rates. AID is expressed by deaths per 100,000 person-years of exposure.[14,15]
**Gini coefficient**[a]	This measure is defined as the average inter-group mortality difference as a percentage of the national mortality rate: $$Gini = \frac{AID}{\overline{SDR}}$$ where $\overline{SDR}$ is the standardized death rate estimated for entire population.[14,15]

[a] Also called as inter-group Gini coefficient or pseudo-Gini coefficient.
